# Supplementary material for: Synthesis, Structural Characterization, Hirschfeld Surface Analysis and Photocatalytic CO2 Reduction Activity of a New Dinuclear Gd(III) Complex with 6-Phenylpyridine-2-Carboxylic Acid and 1,10-Phenanthroline Ligands
Source: Molecules. 2023 Nov 14;28(22):7595. doi: 10.3390/molecules28227595 (PMC10673462; doi:10.3390/molecules28227595)
Supplement: Supplementary file 1 [file molecules-28-07595-s001.zip › molecules-2700649-supplementary.pdf]

Supplementary Material

Synthesis, Structural Characterization, Hirschfeld Surface Analysis and Photocatalytic CO_2 Reduction Activity of a New Dinuclear Gd(III) Complex with 6-Phenylpyridine-2-carboxylic acid and 1,10-Phenanthroline Ligands

Li-Hua Wang [1] and Xi-Shi Tai [2,*]

[1] College of Biology and Oceanography, Weifang University, Weifang 261061, P. R. China
[2] College of Chemistry and Chemical Engineering, Weifang University, Weifang 261061, P. R. China

The synthetic route for complex (1) is shown in Schemes S1.

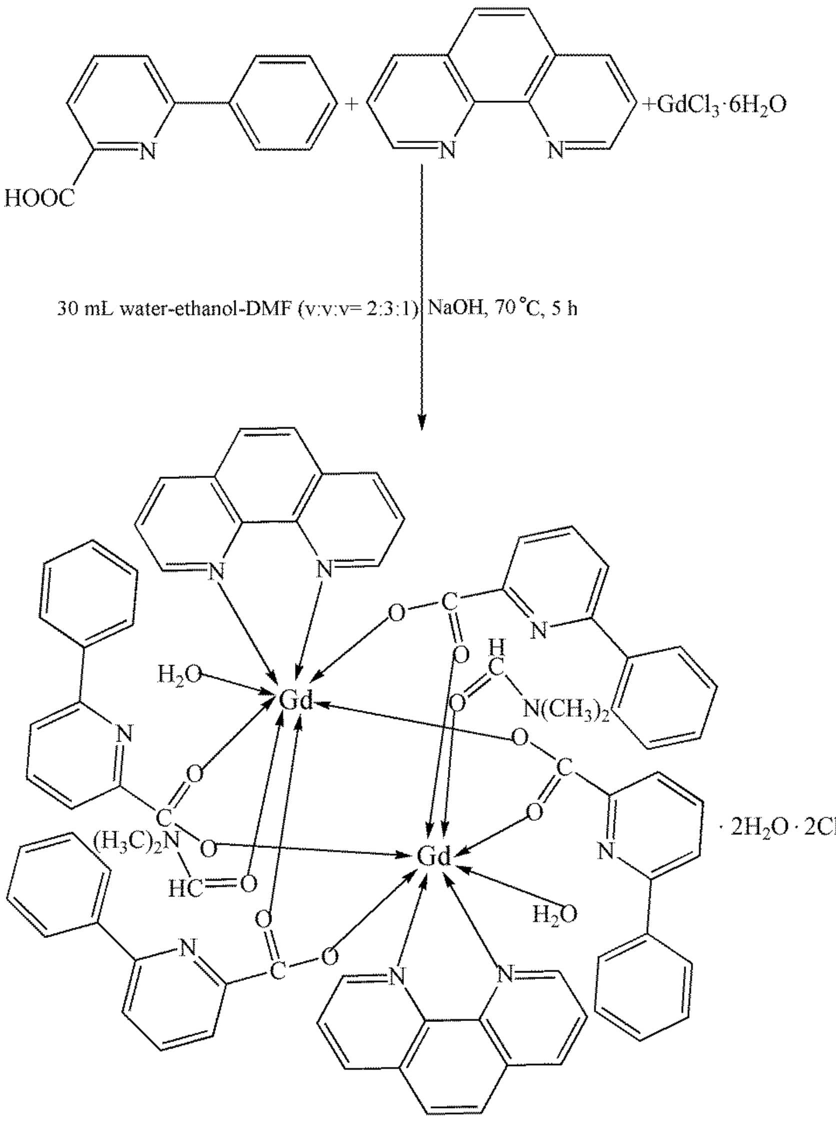

Scheme S1. Synthetic route for complex (1).

The method of the yield calculation is following:

1. mL of the gas has been got out every hour and tested via a gas chromatograph. The peak area of the product was obtained. Subsequently, we quantified the number of moles

of per peak area via using the CO and CH_4 standard gases, which is noted as Y_{1CO} and Y_{1CH4}, respectively. The total amount of CO and CH_4 is calculated according to the equation below.

$$Yco = \frac{Y1co \times Sco \times Sr}{m} \text{ and } Y_{CH_4} = \frac{Y1_{CH4} \times S_{CH4} \times Sr}{m}.$$

Sco and S_{CH4} is the peak areas of CO and CH_4, which was measured using the gas chromatograph. Sr is the reactor volume.

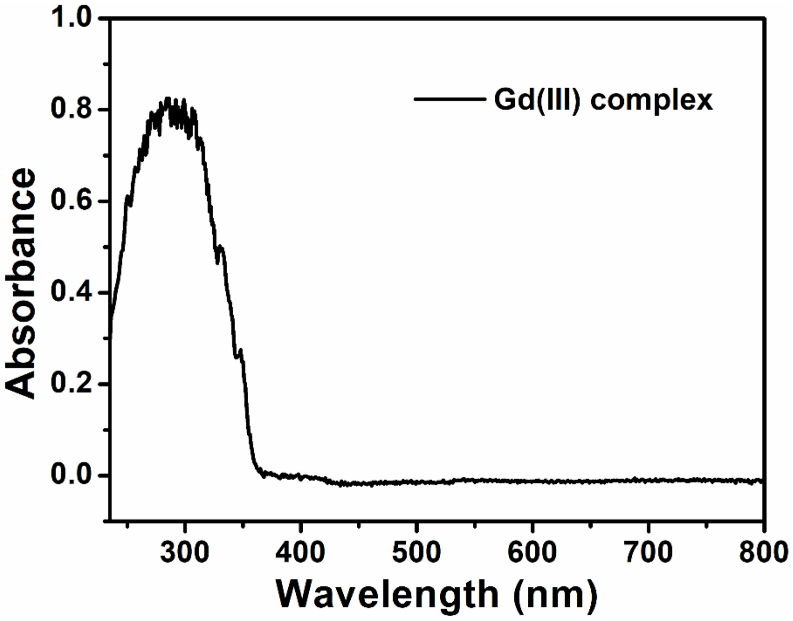

Figure S1. UV-vis absorption spectrum of complex (1).
